# Supplementary material for: Raspberry plant stress detection using hyperspectral imaging
Source: Plant Direct. 2023 Mar 16;7(3):e490. doi: 10.1002/pld3.490 (PMC10020142; doi:10.1002/pld3.490)
Supplement: Supplementary file 1 — Figure S1: Heat maps showing significance of treatment, genotype and interaction effects for biophysical measures at harvest. Blue indicates not significant at 0.05 threshold, teal indicates significant at 0.05 threshold but not 0.01 and yellow indicates significant at 0.01 threshold. Most measures show significant interaction effects. Figure S2: Plots showing the response over time of the ratio r469_523 for different treatments and genotypes. a) lw is low water treatment, b) hw is high water treatment, c) rr is P. rubi treatment and d) vw is vine weevil treatment. A progressive reduction in reflectance response can be seen for Glen Moy in the P. rubi treatment. [file PLD3-7-e490-s003.docx]

Supplementary materials


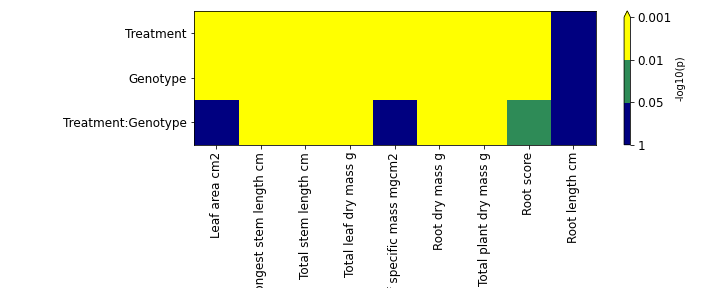


Supplementary figure 1: Heat maps showing significance of treatment, genotype and interaction effects for biophysical measures at harvest. Blue indicates not significant at 0.05 threshold, teal indicates significant at 0.05 threshold but not 0.01 and yellow indicates significant at 0.01 threshold. Most measures show significant interaction effects.


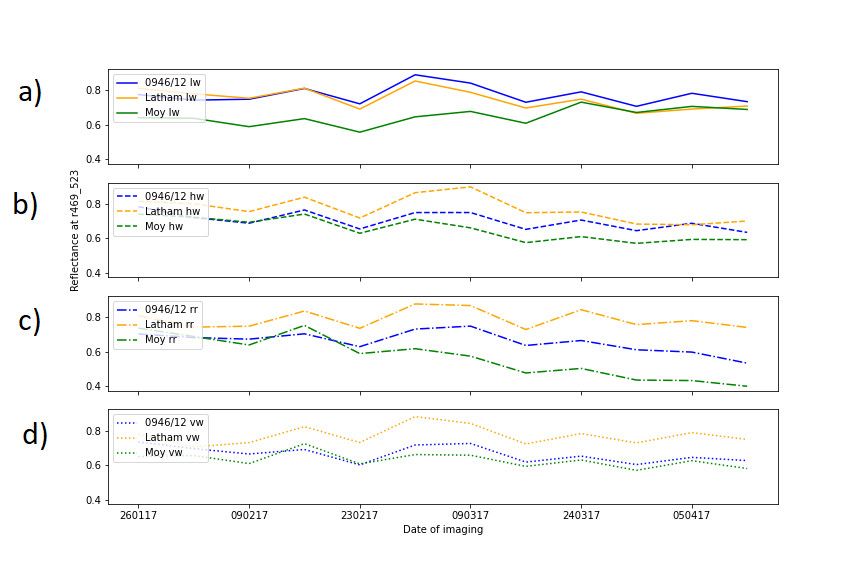


Supplementary figure 2: Plots showing the response over time of the ratio r469_523 for different treatments and genotypes. a) lw is low water treatment, b) hw is high water treatment, c) rr is *P. rubi* treatment and d) vw is vine weevil treatment. A progressive reduction in reflectance response can be seen for Glen Moy in the *P. rubi* treatment.
